# Power-Laws and the Use of Pluripotent Stem Cell Lines

**DOI:** 10.1371/journal.pone.0052068

**Published:** 2013-01-02

**Authors:** Bernhard M. Schuldt, Anke Guhr, Michael Lenz, Sabine Kobold, Ben D. MacArthur, Andreas Schuppert, Peter Löser, Franz-Josef Müller

**Affiliations:** 1 Graduiertenschule Aachen Institute for Advanced Study in Computational Engineering Science (AICES), RWTH Aachen, Aachen, Germany; 2 Robert Koch Institute, Berlin, Germany; 3 Centre for Human Development, Stem Cells and Regeneration, Institute of Developmental Sciences, University of Southampton, Southampton, United Kingdom; 4 School of Mathematics, University of Southampton, Southampton, United Kingdom; 5 Institute for Life Sciences, University of Southampton, United Kingdom; 6 Zentrum für Integrative Psychiatrie, Kiel, Germany; RWTH Aachen University Medical School, Germany

## Abstract

It is widely accepted that the (now reversed) Bush administration’s decision to restrict federal funding for human embryonic stem cell (hESC) research to a few “eligible” hESC lines is responsible for the sustained preferential use of a small subset of hESC lines (principally the H1 and H9 lines) in basic and preclinical research. Yet, international hESC usage patterns, in both permissive and restrictive political environments, do not correlate with a specific type of stem cell policy. Here we conducted a descriptive analysis of hESC line usage and compared the ability of policy-driven processes and collaborative processes inherent to biomedical research to recapitulate global hESC usage patterns. We find that current global hESC usage can be modelled as a cumulative advantage process, independent of restrictive or permissive policy influence, suggesting a primarily innovation-driven (rather than policy-driven) mechanism underlying human pluripotent stem cell usage in preclinical research.

## Introduction

Human embryonic stem cells (hESCs) have attracted much research attention over the last 15 year. Despite of the existence of a steadily increasing number of hESC lines, several studies have noted a preferential use of only a few lines (particularly the WiCell lines derived by Thomson and colleagues in 1998 [1]) in the vast majority of hESC studies, both within the United States (US) and throughout the rest of the world [2]–[6], although regional differences in stem cell usage patterns have been reported [5].

In 2001 the Bush administration decided to restrict federal funding through the National Institutes of Health (NIH) to research using hESC lines derived prior to August 9^th^ of that year (there are 21 such lines; we will refer to them as “formerly NIH approved hESC lines”). This policy decision has stirred much controversy [7], [8] and has been perceived as providing a long-lasting impediment for competitive hESC research in the US by providing a restrictive bias towards the use of formerly NIH approved cell lines [9]–[11]. Although President Obama lifted the Bush administration’s restrictive policy in 2009 [12] (and despite the fact that several-state run research programs have been created in the last decade to facilitate more diverse hESC research [13]), the on-going preferential use of a subset of the Bush-approved cell lines has also primarily been attributed to the NIH’s funding policy.

However, the scientific basis for these claims remains obscure [3], [4], [6], [14]. In particular, the policy-driven model does not adequately explain the preferential usage of a small subset of the formerly NIH-approved hESC lines (particularly, WiCell’s H1 and H9 lines); nor does it adequately account for the similar hESC usage patterns observed in countries with different (including diametrically opposing) stem cell usage policies. Motivated by these apparent inconsistencies, we set out to understand the generative mechanisms underlying global hESC usage.

**Figure 1:**
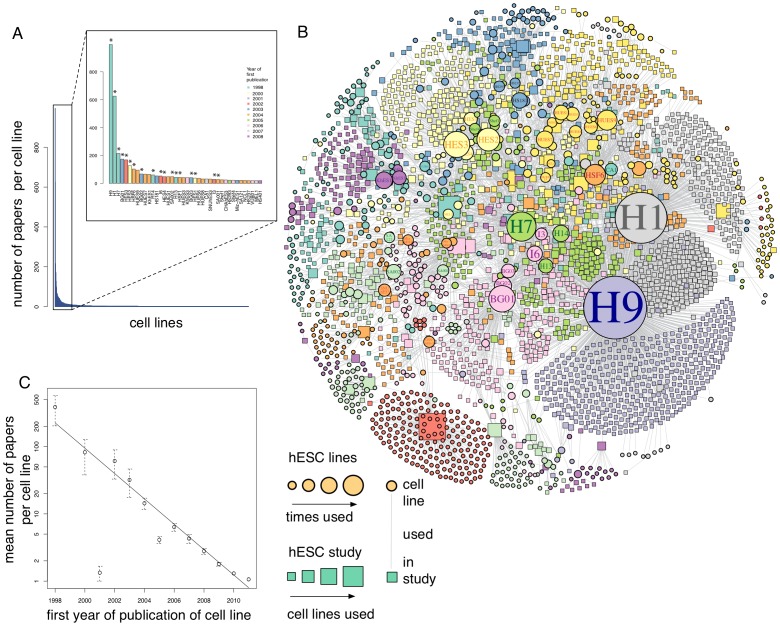
Analysis of the hESC co-citation network. (**A**) Frequency distribution for hESC line use based on the evaluation of 2,338 studies reporting original research involving hESCs and published in peer-reviewed English language journals from 1998 to 2011. The inset highlights the 40 most used lines. Asterisks denote those hESC lines available and eligible for federal funding under the Bush administration from Aug. 9, 2001 to Mar. 9, 2009. Note that in most papers several hESC lines were used. (**B**) The largest connected network of the empirical hESC co-citation network. Peer reviewed studies involving experiments with identifiable hESC lines published from 1998 to 2011 are represented as boxes; hESC lines are represented as circles (see **Fig. S2** for the entire network, including all disconnected components). The network is dominated by few lines (H1, H7, H9, HES-2, HES-3, BG01), which were introduced early in the stem cell field. (**C**) Correlation of hESC usage with time of derivation. Lines derived earlier are used more frequently than those lines derived later, a pattern that appears to be independent of policy influence. Error bars: standard deviation of mean.

We analysed more than 2,300 peer-reviewed studies that documented the experimental use of identifiable hESC lines. Thus, we chose to study the actual use of hESC lines in successfully completed basic and preclinical research projects. This is in contrast to other studies, which have considered a variety of proxies for actual hESC usage including: material transfer agreements on shipping of cell lines from selected providers [3], [4]; preliminary results presented at a single conference [6]; data on the intended hESC usage in grant applications [13], [15]; and information obtained from surveying stem cell scientists in the US [16], [17]. Analysis of our data indicated a striking heavy-tailed distribution of hESC usage, as has been previously observed [16], with most studies only making use of a small number of hESC lines (**Fig. 1A**). To interpret this data we compared the ability of a policy-driven model and a simple cumulative-advantage model, based upon dissemination of cell lines within an evolving scientific collaboration network, to explain hESC usage. We find that current hESC usage patterns can be easily and more precisely explained by a policy-independent model.

## Materials and Methods

### Literature Search

Database searches for hESC research papers were performed as previously reported [5]. Our initial search resulted in more than 11,000 hits for papers listed in the PubMed database and published in peer-reviewed English language journals through the end of 2011. Criteria for paper extraction as well as for assignment of papers to specific countries are reported elsewhere [5]. Briefly, papers were manually evaluated to exclude those manuscripts in which hESCs were not used experimentally (e.g. commentaries, reviews, news, and editorial articles; work on mouse embryonic stem cells or human embryonic carcinoma cells; papers on ethical or political aspects of hESC research etc.). Articles that summarized previously described methods and protocols as well as those in which only hESC-derived materials (but not hESCs themselves) were used were also excluded. In total we found 2,403 primary research articles that reported the derivation and/or experimental use of hESCs. Of these 2,403 original research papers, 65 (2.7%) did not contain sufficient details concerning the specific hESC cell lines to be used and were therefore also excluded from further analysis. In total we therefore considered data from 2,338 unique research articles. A number of primary cell lines (such as H9, H1, AS034 and HES-3) were represented by several secondary sub-lines [e.g. H9.1, H9.2, H1.1, H1-OGN, AS034.1, HES-3.gfp (ENVY)]. These secondary sub-lines were collapsed into the parental lines (e.g. H9.1, H9.2 etc. were all considered H9). Funding information was retrieved from the appropriate sections in the paper text.

Database searches for papers reporting derivation and experimental use of human induced pluripotent stem cells (hiPSCs) were performed according to reference [18] and resulted in a set of 514 research papers published through the end of 2011. Criteria for inclusion of a paper were basically the same as for hESC research papers.

Assignment of papers to specific countries was performed according to the academic affiliation of the corresponding author. A detailed paper list is available on request.

### Network Simulation

We compared our empirical findings with simulations of a Yule-Simon process [19], [20]: a model commonly used for simulations of social networks. We adapted the original Yule-Simon process [19], [20] using a procedure proposed by Morris for bipartite graphs [21].

In our simulations we assumed that all studies use the same number of hESC lines (N = 2). We selected this parameter since it is close to the observed mean number of cell lines used per study in the main connected component of the empirical hESC usage network (in the empirical network, the mean number of lines used per publication is 2.47). To compensate for these minor differences we increased the number of studies in our simulation to 2,927 to arrive at the same overall number of citations.

**Figure 2:**
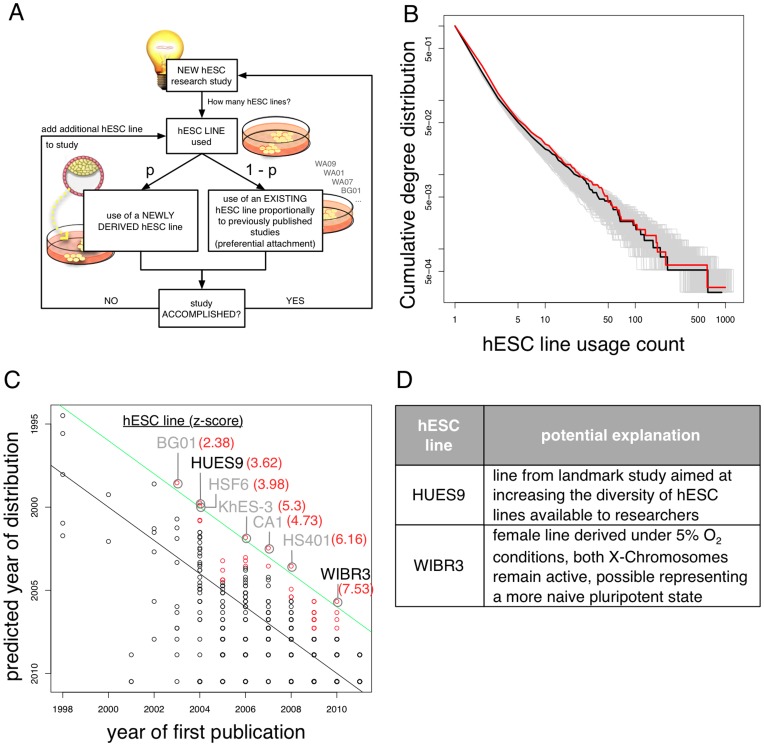
A cumulative advantage model for global hESC usage patterns. (**A**) Schematic of our simple cumulative advantage model. (**B**) The observed cumulative frequency distribution of hESC usage is in red; 1000 simulations of the model outlined in (A) are shown in grey. For illustrative purposes, the best fit of the model to the data is shown in black. (**C**) Policy-independent assumptions allow for the statistical detection of “anomalous” lines in the empirical data (all lines represented by circles). A predicted distribution year for each of the published 995 cell lines, based on their usage patterns, was computed and the predicted year was compared with the actual first publication year. A predicted distribution date strikingly earlier than the actual publication date indicates that a published line is more widely used than would be expected by comparison with those lines published around the same time. The green line separates from the bulk population those lines with a predicted distribution date at least 4 years earlier than their actual publication date. Seven hESC lines (z-scores in red) appear to have been distributed at least four years earlier than their actual publication date. Five of these lines (BG01, HSF6, CA1, KhES-3 and HS401; shown in grey) were in fact derived and distributed significantly before their first peer reviewed publication date (see **Table S4**). Lines used significantly more often than others published in the same year (z-score >2) are indicated in red. (D) Possible technological explanations for the observed unusual usage patterns of hESC lines HUES9 and WIBR3.

In our simulations (see **Fig. 2B**), when a new stem cell study is started, we decide how many novel hESC lines are introduced based on a binomial probability of success in *N* independent draws with an associated probability *p*. The newly generated stem cell study is then added to the stem cell research network. If a study does not use a novel line, then the lines employed in the study are chosen from the preexisting cell lines already in use. We took the probability of selecting an individual line from the pool of preexisting hESC lines to be proportional to the number of times each cell line has been previously used in other studies. To estimate the probability with which a novel line is chosen, we ran our model with probabilities of 0.05, 0.1, 0.15, 0.2, 0.25 and 0.3 for introducing a new hESC line into the simulated network. We selected a probability of 0.15 based on comparison with the empirical dataset. The resulting networks are directed bipartite graphs, which are not necessarily connected. In order to obtain statistics of usage we generated 1,000 random simulated instances of our model.

We used the power.law.fit method from the igraph package with xmin = 3 to estimate the exponent for the in-degree distribution of the network [22]. Although we find a good fit we caution that our empirical hESC co-citation network only contains 2,338 nodes, which is relatively small for reliable direct parameter estimation. Direct methods for determining fit to power-laws usually require datasets with several orders of magnitude more observations; we refer the reader to Clauset and Newman [23], [24] for a comprehensive discussion of the challenges and methods involved in estimating power-law distributions from empirical data. The R script of our simulation implementation is available on request.

### Network Analysis

We first analyzed the connected components of the networks. The biggest component contains the largest number of publications. We found the remaining components were dominated by cell lines that were used only in a single study and often appeared in manuscripts that reported the derivation of a large number of new hESC lines. We conclude that the biggest component reflects most hESC research activity. Empirical network analysis and visualization was performed in R as in reference [25] using the igraph [22] package. Additional plots were generated with Cytoscape [26].

### Hierarchical Clustering

cluster global usage we selected only those countries with at least 30 publications to avoid spurious effects due to small sample sizes. The distances between the 14 identified countries were calculated as one minus the Spearman correlation of the hESC usage patterns. Hierarchical clustering was then performed in Matlab (Mathworks, Natick, MA, USA) using average linkage. The assignment of a nationality to study was based on the affiliation of the corresponding author.

### Stem Cell Usage and Time of First Publication

For each cell line, the number of studies it was used in and the year of first publication were determined. A linear regression model using the year of first publication as the independent variable and the logarithm of usage frequency as the dependent variable was calculated using robust linear regression in R using the MASS package [27]. The resulting regression model was used to predict the year of first publication depending on the frequency of usage **(** **Fig. 2C** **)**. The z-score was calculated based on the mean and standard deviation of all cell lines that were first published in the same year.

## Results

We first analysed the usage of hESC lines in 2,338 peer-reviewed studies that reported original experimental research using identifiable hESC lines published worldwide through the end of 2011. This data confirmed the continuing preferential use of a few prominent hESC lines derived early in the history of the field (**Fig. 1A and B**). This is in agreement with previous observations made by us and other groups [2]–[6], [13], [15], and occurs despite the existence of a considerable and diverse library of more than 1,000 hESC lines (as of the end of 2009) for the research community to use [5]. We note that even among the 21 former eligible lines – of which 18 were distributed by the same stem cell bank – usage frequency varies considerably (from H9 used in 996 studies; to I4 used in 8 studies, **Fig. 1A**).

We found no correlation between national hESC policy (i.e. restrictive versus permissive) and the use of specific hESC lines (**Fig. S1)**. Rather, we found that hESC use correlates strongly with year of derivation (**Fig. 1C**). Furthermore, we observed that the frequency distribution for hESC use could be approximated by a power-law (**Fig. 1A**). Power-law like distributions of influence have been observed in many complex systems, particularly in citation and social networks [23], [28]. In these networks, power-law like distributions are often assumed to result from an underlying generative mechanism that has been termed in different contexts a *Matthew effect, Yule process, cumulative advantage* or *preferential attachment* [23], [28]. In this model, a small number of initial “founder” nodes gain a disproportionate share of the interactions in the network through a “rich-get-richer” process as the network grows.

Given these parallels we asked whether similar mathematical models could equally well explain why specific subsets of hESC lines are used preferentially in the US and worldwide. We hypothesized that the emergence of the observed distribution of hESC usage, skewed towards the frequent use of only very few cell lines derived early on, could be modelled as a cumulative advantage process without the need for additional mechanisms such as the influence of funding policies or legal restrictions.

To do this we constructed a simple mathematical model (outlined schematically in **Fig. 2A**) based on the assumption that published peer-reviewed experimental studies involving hESC lines accurately serve as a surrogate to assess the use of hESC lines in specific hESC research projects. To start the model we assumed that a few founder lines are generated in a first project and were made available to a broader research community (as is the case for hESC research) [1]. Next we assumed that new hESC studies are performed over time, with each new study using two distinct hESC lines (this is in agreement with the observed empirical average of 2.47 hESC lines per study, see Materials and Methods). In each new study, we assumed that new hESC lines are derived with probability *p* or, alternatively, pre-existing lines are used with a probability 1-*p*. In accordance with a cumulative advantage process we assumed that the probability of selecting an individual pre-existing hESC line is proportional to the number of pre-existing studies already using that line. This is plausible since stem cell lines with efficient distribution channels; those which already exist in the freezers of a research institution; or those for which there is a wealth of experience and published data are likely to be preferentially chosen when planning new experiments. This simple model results in simulations of a growing hESC usage network. This network is a bipartite graph with stem cell lines and published studies as nodes, in which there is an edge between a cell line and a study if the study uses that cell line. The frequency distribution for the use of hESCs in research may then be obtained by counting the number of edges between publications and cell lines. Crucially, in this model, the current status of hESC usage is dependent upon the history of hESC usage. Particularly this model strongly favours hESC lines that were introduced early (a phenomenon known as the “first mover advantage” in scientific co-citation networks) [24], [29] (**Fig. S3C**). It should be pointed out that our model does not contain a discernible “policy term”, which could be interpreted as governmental “power” driving stem cell research through laws or regulations towards the usage of only few “eligible” lines**.**

To compare empirical data with our model we also generated an empirical hESC usage network. As with the simulated networks, this network is a bipartite graph with stem cell lines and published studies as nodes, in which there is an edge between a cell line and a study if the study uses that cell line. (**Fig. 1B** **, Fig. S2**). We find that our cumulative advantage model (**Fig. 2A**) resulted in simulated networks strikingly similar to the observed empirical hESC usage network (**Fig. 2B** **, Fig. S3)**.

To further analyse at least anecdotal evidence for a policy-driven model, we examined the interesting case of the California Institute for Regenerative Medicine (CIRM): a multi-billion dollar research organisation established, in part, to counteract the possibly negative effects of political funding restrictions, and enable California-based researchers to derive and use cell lines other than those eligible for NIH funding during the Bush presidency [30], [31]. We reasoned that, assuming a policy-driven model, one would expect at least a trend towards the increased use of those hESC lines *not* eligible for NIH funding in CIRM-associated publications. However, we found that of 185 hESC studies bankrolled at least in part by CIRM and published from 2008 to 2011, 171 (92.4%) used prominent formerly NIH approved hESC lines introduced early in the field (**Table S1**). While 28 (15.1%) used other hESC lines in addition to formerly NIH approved lines, 143 CIRM-funded studies (77.3%) were exclusively based on formerly NIH approved hESC lines. Only 14 studies (7.6%) were performed without use of any formerly NIH approved hESC lines. Since 110 of the 185 CIRM-funded studies were also supported by NIH grants, we investigated more closely those 75 CIRM funded studies that were performed without additional NIH funding. In 63 (84%) of these studies, at least one formerly NIH approved hESC line was used, and in 50 studies (66.7%) formerly NIH approved hESC lines were used exclusively. Only 12 studies (16%) were performed without the use of any formerly NIH approved hESC line. This is in accordance with recent analyses of state-funded stem cell research grants, including those from CIRM, which have suggested a similar preference for formerly NIH approved lines at the grant application stage of the research process [13], [15]. We also observed a comparable pattern of hESC line usage in studies from Germany after strict regulations, similar to those in the US, were significantly lifted in 2008 (**Table S2**).

**Figure 3:**
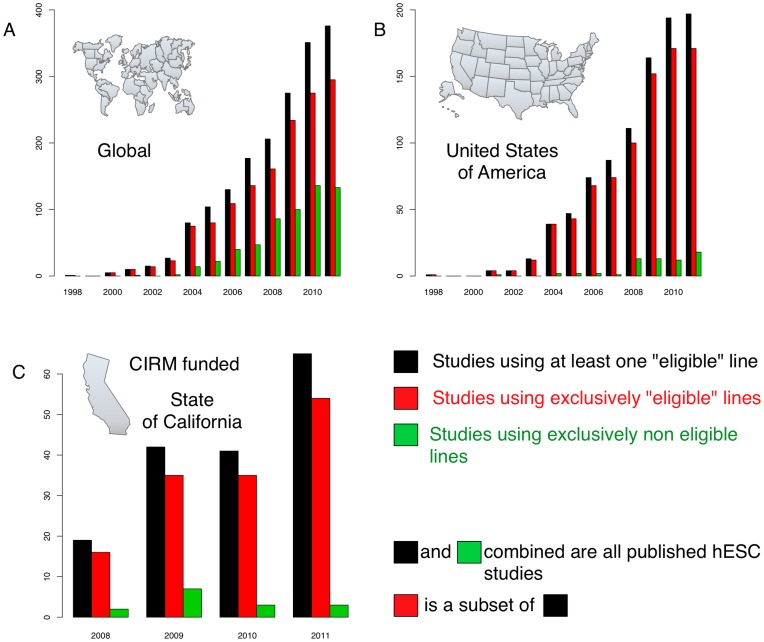
Worldwide usage patterns of hESC lines. Changes over time in the number of studies using eligible (formerly NIH approved) and non-eligible hESC lines is shown. Panel (A) shows global patterns; (B) patterns in the US and; (C) patterns in CIRM funded studies. Although the Bush administration’s restrictions on hESC usage were lifted in March 2009 the number of publications exclusively using formerly restricted lines did not increase significantly in any region from 2009–2011. For a detailed statistical comparison of these data see **Table S3**.

To investigate this further we performed a hypergeometric test to determine if there are any statistically significant differences in use of formerly NIH-approved hESC lines in CIRM-funded, NIH-funded and worldwide studies. We found that in both the US and worldwide there was a significant preference for the use of early derived formerly NIH approved lines (**Fig. 3**, **Table S3**). This was also true for CIRM funded studies, despite independent funding. These results indicate a firm attachment to early hESC lines, particularly those derived in the US by Thomson and colleagues in 1998 [1].

To investigate further if stem cell usage might be more independent of policy decisions than widely assumed, we investigated cell line usage in the rapidly developing, and far less regulated, field of human induced pluripotent stem cell (hiPSC) research. Thomson et al. [32], Yamanaka et al. [33], [34] and Daley et al. [35] derived the first hiPSC lines in 2007. hiPSCs are widely believed to represent a seminal breakthrough in stem cell research [36]. Although there are still open questions regarding qualitative differences between hiPSCs and hESCs [37], they potentially provide an inexpensive, robust and ethically less controversial means to derive patient-matched human pluripotent cells. Among the 512 original research papers involving hiPSCs published from 2008 through the end of 2011 we identified 201 manuscripts that used previously established hiPSC lines in which the lab of origin is clearly specified. Due to lack of a consistently used cell line nomenclature, it is very difficult to reliably track the use of individual hiPSC lines [38]. Thus, we used the lab provenance as a proxy measure for actual hiPSC usage. Of the aforementioned 201 studies, 54 (26.9%) used hiPSC lines reported by the Thomson lab in 2007 [32], 29 (14.4%) used cell lines reported by Yamanaka and colleagues [33], [34] and 28 (13.9%) used hiPSCs developed by Daley and co-workers [35]. The tendency to the preferred usage of only few hiPSC cell lines is not as pronounced as in the case of hESCs. hiPSCs are more easily derived than hESCs and many groups have produced their own hiPSC lines for use in multiple studies. Nevertheless, the share of papers using those first hiPSC cell lines published in 2007 increases to about 70% if only those 93 studies in which investigators did not possess their own hiPSCs (but exclusively used lines derived in other laboratories) are taken into account. The fact that early hiPSC lines derived in only three labs are employed in more than 50% of hiPSC studies using previously established lines suggests that a first-mover effect may also be an emerging property in the hiPSC research field.

We finally asked if our cumulative advantage model could be useful for identifying emerging hESC usage trends from empirical data. To do this we fitted a regression model to the observed hESC usage distribution in order to compare actual hESC first publication dates with predicted hESC distribution dates based solely on observed usage frequencies per year. We combined this method with a z-score procedure previously proposed for the identification of outliers (in our case, individual hESC lines) [24]. On the basis this analysis we tentatively predict that later established yet highly utilized hESC lines, may have been widely distributed before the date of their first publication (i.e. they are “older” than they appear) or, more interestingly, might be technologically more relevant to future research than the remaining bulk of hESC lines of comparable age. Using this method we highlighted 7 widely used but later-derived hESC lines of interest, out of a total of 955 (**Fig. 2** **C and D, Table S4**). Five of these lines (BG01, HSF6, HS401, CA1, KhES-3) were indeed derived and distributed significantly before their first peer reviewed publication date (see **Table S4**). The remaining two lines (HUES9 and WIBR3) illustrate two longstanding innovation trends in hESC research: 1) the HUES9 line originates from the Melton lab [39] and exemplifies the push towards diversifying available hESC lines, and 2) the WIBR3 line examplifies ongoing efforts to derive more naïve hESC lines [40].

## Discussion

In this study, we analysed human pluripotent stem cell usage patterns by investigating a comprehensive dataset based on published hESC research and proposed a simple mathematical model that qualitatively explains hESC usage patterns. Our analysis shows that a simple first-mover generative mechanism, that does not rely on funding policy as a causative factor, can naturally explain the observed dominance in use of a small number of hESC lines. Although our analysis does not exclude the influence of policy decisions on hESC usage, the results question accepted wisdom connecting stem cell usage patterns exclusively to US funding policies [3], [4], [6], [14].

In agreement with a purely policy-driven interpretation of hESC usage, our model favours a set of early “founder” hESC lines. However, in our model preferential use of these lines is not due to political restrictions but rather arises naturally due to collaborative processes within the scientific community. We argue that if hESC use is a purely policy-driven process then qualitatively different patterns of usage of “approved” and “restricted” cell lines should be apparent in differing political environments. However, when analysing worldwide usage patterns we observed no such distinctions (**Fig. 1A and B**), suggesting that alternative underlying generative mechanisms significantly contribute to observed hESC usage patterns.

Several issues are associated with fitting power laws to empirical datasets (particularly those with relatively small statistical support) and the cumulative advantage process used here is only one of several possible models that could have been applied [41]. Consequently, we caution that our proposed model may represent just one possible alternative to a policy-driven interpretation of hESC usage patterns: rejecting one hypothesis over another in a complex process such as global hESC research is difficult on purely statistical grounds, particularly using data from a relatively small sample of 2,338 publications. Nevertheless, our model indicates that disruptive innovation (i.e. breakthroughs which fundamentally change the research landscape, such as the derivation of the first hESC lines) and sustaining innovation (i.e. refinements to existing hESC derivation and culture protocols) may be primary drivers of worldwide hESC usage.

While much effort has led to improvements in hESC lines and cultures since they were first derived by Thomson and colleagues [1], their basic properties (i.e. self-renewal and multi-lineage differentiation potential) remain unchanged, as do the challenges (i.e. ethical problems) associated with their production. Even though ethically less problematic lines from single blastomeres [42] or biologically more naïve hESC [40] have been subsequently established, these lines have not been nearly so well used as the earliest lines derived. By the end of 2009 publically available information on more than 1,000 hESC lines was available from the scientific literature, stem cell registries and banks, press releases and institutional webpages [5]. Since then, the number of hESC lines has further increased to nearly 1,600 as of July 2012. These hESC lines have been derived for a number of reasons, including: to better understand the derivation process, for example by varying derivation conditions [43] or by using poor quality, arrested or aneuploid embryos [44]–[46] as a source for hESCs; to obtain cell lines with particular monogenetic disorders in order to create disease-specific hESC based cell models [47]; and to obtain clinical-grade hESC lines by performing derivation under xeno-free conditions [48]. In a single study from China nearly 200 hESC lines were produced to match a broad panel of HLA phenotypes [49]. Additionally, the prospect of creating “national” banks of hESC lines may also contribute to the drive to diversify [50], [51]. However, of the nearly 1,600 hESC lines publically known by July 2012, about 500 have been not, as yet, been reported in the scientific literature; while another approximately 650 have been used in only one report. The relevance of novel hESC lines may therefore lie not in their frequency of use, but rather in their value as assets in the clinical development portfolio of organizations aiming at advancing hESC-based therapies (such as CIRM and the NIH), as well as in the IP portfolio of biotechnology companies such as Advanced Cell Technology Inc.

Our outlier analysis (**Fig. 2C and D**) indicates that technological, sustaining innovation is a key driving-factor underlying the derivation of such novel cell lines. The enormous resources and investments that went into the development of clinical grade hESC lines reveal a weakness of our (and others’) publication-based analyses. On a global scale, development of such cell lines will continue to merely increase the long tail of the observable hESC usage distribution in spite of potentially low thresholds to access to independent funding. Consequently, we predict that most basic and preclinical research projects will continue to choose the most frequently used and cited hESC lines as a starting point for their research and clinical grade stem cell lines, such as HS401, will remain under-utilized in preclinical hESC studies (although, in the long-run the collection and dissemination of a wide panel of reliable data for many hESC lines by stem cell registries and banking initiatives [52]–[54] may contribute to the diversification of hESC usage). It remains to be seen if such latter-derived (and infrequently used) yet technologically innovative lines will ultimately come to dominate clinical applications. The first hESC-derived cells to be transplanted into a patient were differentiated from the H1 hESC line [55], one of the first lines reported by Thomson and co-workers in 1998 [1]. The H1 line is also one of the two most widely used lines in the preclinical stem cell field (**Fig. 1**) and is predicted by our model to remain so. However, the same formerly NIH approved cell line received much attention in 2006 as it was found to be contaminated with the nonhuman sialic acid Neu5Gc in animal component derived culture systems [56], although improved xeno-free culture techniques have obviously rendered this technical problem solvable for NIH approved clinical trials [55].

Taken together, our analyses question a substantial impact of the Bush policy on use of specific hESC lines in basic and preclinical research both globally and in the US. We conclude that policy decisions concerning present and future funding for both hESC and hiPSC research should account for alternative generative mechanisms underlying human pluripotent stem cell usage.

## Supporting Information

Figure S1 **Hierarchical clustering of global stem cell usage patterns.** Nations with at least 30 experimental hESC publications were included in the analysis (AU: Australia, CA: Canada, CN: China, DE: Germany, ES: Spain, FI: Finland, FR: France, GB: United Kingdom, IL: Israel, JP: Japan, KR: South Korea, SE: Sweden, SG: Singapore, US: United States of America). Spearman rank correlation was used to assess similarity in hESC usage patterns (see **Materials and Methods**). When assigning a study to a country we used the institutional affiliation of the corresponding author. Countries with diametrically opposing policies on hESC use cluster together despite regulatory differences. The policy classification for 2005 is according to reference [57]; while that of 2012 was retrieved from http://mbbnet.umn.edu. Note that the displayed policy classification is only a relatively coarse-grained measure for complex national hESC usage regulations.
(TIF)

Figure S2 **Global hESC co-citation network.** Full hESC co-citation, including all peer-reviewed studies involving experiments with identifiable hESC lines published from 1998 to 2011. The network is a bipartite graph in which both hESC lines and studies are drawn as circles. There is an edge between a cell line and a study if the study uses that cell line. Most studies belong to the largest connected component (in blue). Several considerably smaller components (in orange) contain work with hESC lines that rarely appear in other publications. The main component network is displayed in more detail in Fig. 1B.
(TIF)

Figure S3 **Observed and simulated network parameters.** We simulated a cumulative advantage process for the usage of stem cell lines (as shown in **Fig. 2B** and described in the **Online Methods)** 1,000 times and compared the parameter distributions with the actual observed data points (marked red). **(A)** Histogram of the distance (area enclosed between the curves) between each simulation of our model and the empirically observed stem cell usage data. The simulated result with the smallest distance to the observed usage data is given in black in **Fig. 2B**. **(B)** Histogram of estimated power law exponents for each simulation of our model. The estimated empirical exponent (1.94) is shown as a red line**. (C)** The frequency with which “founder” lines (the first 5 simulated lines in each simulation) and subsequent lines were used in simulated networks. **(D)** Histogram of the maximum number of citations received by a cell line in our simulations (i.e. the maximum node degree in the final simulated networks). The value observed for the empirical hESC co-citation network (the H9 line which received 996 citations) is shown in red line. **(E)** Histogram of the number of hESC lines in the largest connected component of the simulated co-citation networks. The value observed for the empirical hESC co-citation network (794) is shown in red.
(TIF)

Table S1 **Use of hESC lines in studies (co-)funded by the California Institute for Regenerative Medicine.** Statistics of hESC use for all published hESC studies with CIRM-funding (including those that also received additional funding from other sources, e.g. from the NIH) are shown. CIRM funding started in 2006, so studies published from 2008 were investigated. Funding information was taken from the appropriate section of the papers. The percentage share in the total number of papers published is in brackets. Note that more than one hESC line is used in most studies; thus the percentages add up to more than 100.
(DOCX)

Table S2 **Use of hESC lines in research papers from Germany.** Only cell lines that were used in at least two studies are listed. Numbers in brackets indicate the percentage share of the 56 hESC research papers from Germany published through the end of 2011. Of all the hESC lines that became accessible to German scientists after the amendment of the German Stem Cell Act in 2008, only one (HUES2) was used in more than one published study (note that prior to 2008 use of hESC lines in Germany was restricted in principal to those lines formerly approved by the NIH). In most studies more than one hESC line is used; thus the percentages add up to more than 100.
(DOCX)

Table S3
**Enrichment analysis for formerly NIH approved hESC lines.** Statistically significant differences in use of formerly NIH-approved hESC lines by comparison with other hESC lines were identified using hypergeometric enrichment analysis. Significant *p*-values indicate enrichment of use of the cell lines indicated in the column headers. We observe a highly enriched usage of the formerly NIH approved hESC lines in the US both before and after policy changes in 2009. This pattern is repeated in CIRM funded studies. We note a trend in the CIRM subset towards an increased number of studies using exclusively non eligible lines (i. e. other lines than the formerly approved NIH hESC lines). 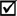 indicates significant difference; 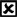 indicates no significant difference.(DOCX)

Table S4 **Characteristics of the seven hESC lines highlighted in** **Fig. 2C and D** **.**
(DOCX)
