# Supplementary figures and images for: Power-Laws and the Use of Pluripotent Stem Cell Lines
Source: PLoS One. 2013 Jan 2;8(1):e52068. doi: 10.1371/journal.pone.0052068 (PMC3534668; doi:10.1371/journal.pone.0052068)

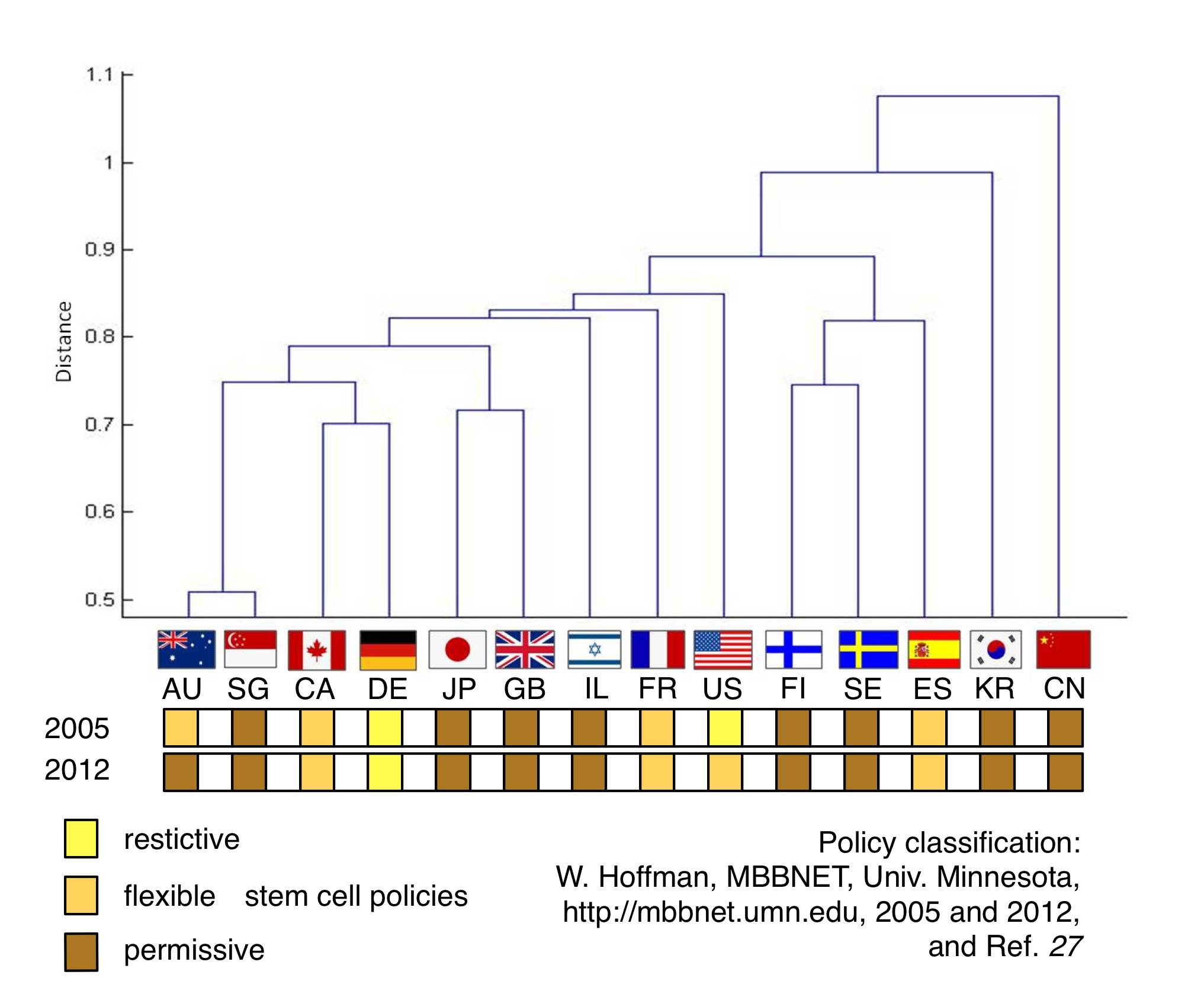

Supplement: Figure S1 — Hierarchical clustering of global stem cell usage patterns. Nations with at least 30 experimental hESC publications were included in the analysis (AU: Australia, CA: Canada, CN: China, DE: Germany, ES: Spain, FI: Finland, FR: France, GB: United Kingdom, IL: Israel, JP: Japan, KR: South Korea, SE: Sweden, SG: Singapore, US: United States of America). Spearman rank correlation was used to assess similarity in hESC usage patterns (see Materials and Methods). When assigning a study to a country we used the institutional affiliation of the corresponding author. Countries with diametrically opposing policies on hESC use cluster together despite regulatory differences. The policy classification for 2005 is according to reference [57]; while that of 2012 was retrieved from http://mbbnet.umn.edu. Note that the displayed policy classification is only a relatively coarse-grained measure for complex national hESC usage regulations. (TIF) [file pone.0052068.s001.tif]

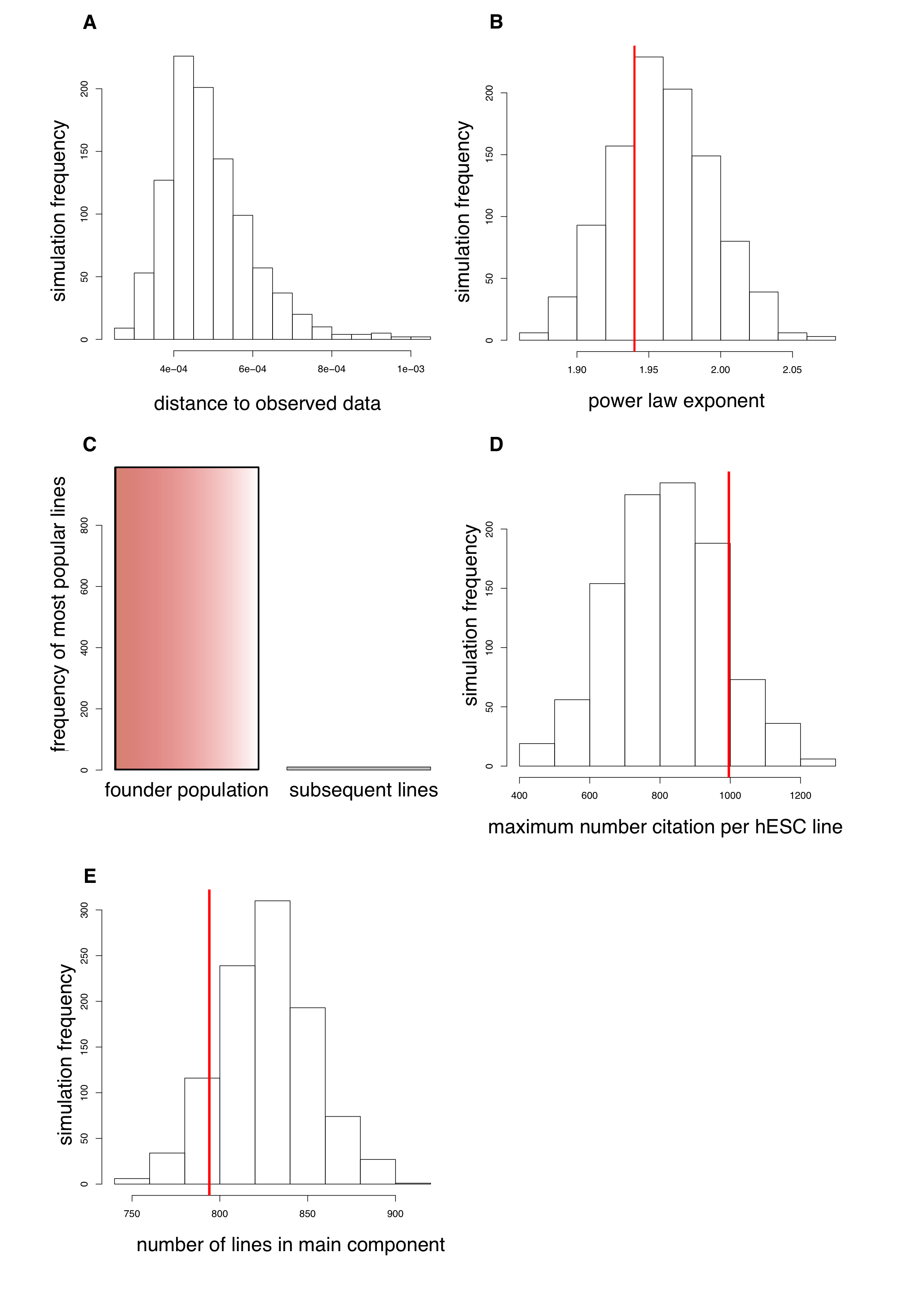

Supplement: Figure S3 — Observed and simulated network parameters. We simulated a cumulative advantage process for the usage of stem cell lines (as shown in Fig. 2B and described in the Online Methods) 1,000 times and compared the parameter distributions with the actual observed data points (marked red). (A) Histogram of the distance (area enclosed between the curves) between each simulation of our model and the empirically observed stem cell usage data. The simulated result with the smallest distance to the observed usage data is given in black in Fig. 2B . (B) Histogram of estimated power law exponents for each simulation of our model. The estimated empirical exponent (1.94) is shown as a red line. (C) The frequency with which “founder” lines (the first 5 simulated lines in each simulation) and subsequent lines were used in simulated networks. (D) Histogram of the maximum number of citations received by a cell line in our simulations (i.e. the maximum node degree in the final simulated networks). The value observed for the empirical hESC co-citation network (the H9 line which received 996 citations) is shown in red line. (E) Histogram of the number of hESC lines in the largest connected component of the simulated co-citation networks. The value observed for the empirical hESC co-citation network (794) is shown in red. (TIF) [file pone.0052068.s003.tif]
