# Supplementary material for: Power-Laws and the Use of Pluripotent Stem Cell Lines
Source: PLoS One. 2013 Jan 2;8(1):e52068. doi: 10.1371/journal.pone.0052068 (PMC3534668; doi:10.1371/journal.pone.0052068)
Supplement: Table S1 — Use of hESC lines in studies (co-)funded by the California Institute for Regenerative Medicine. Statistics of hESC use for all published hESC studies with CIRM-funding (including those that also received additional funding from other sources, e.g. from the NIH) are shown. CIRM funding started in 2006, so studies published from 2008 were investigated. Funding information was taken from the appropriate section of the papers. The percentage share in the total number of papers published is in brackets. Note that more than one hESC line is used in most studies; thus the percentages add up to more than 100. (DOCX) [file pone.0052068.s004.docx]

**Table S1. Use of hESC lines in studies (co-)funded by the California Institute for Regenerative Medicine.**

Statistics of hESC use for all published hESC studies with CIRM-funding (including those that also received additional funding from other sources, e.g. from the NIH) are shown. CIRM funding started in 2006, so studies published from 2008 were investigated. Funding information was taken from the appropriate section of the papers. The percentage share in the total number of papers published is in brackets. Note that more than one hESC line is used in most studies; thus the percentages add up to more than 100.

| Period | Total number of hESC research papers | Number which used at least one “eligible” hESC line | Number which exclusively used “eligible” hESC lines | Number which exclusively used WiCell lines |
| --- | --- | --- | --- | --- |
| **All studies with CIRM funding** | | | | |
| 2008 | 22 | 20 (90.9 %) | 17 (77.2 %) | 12 (54.5 %) |
| 2009 | 51 | 44 (86.3 %) | 37 (72.5 %) | 27 (52.9 %) |
| 2010 | 44 | 41 (93.2 %) | 35 (79.5 %) | 26 (59.1 %) |
| 2011 | 68 | 66 (97.1 %) | 54 (79.4 %) | 40 (58.8 %) |
| **Total (2008-2011)** | **185** | **171 (92.4 %)** | **143 (77.3 %)** | **105 (56.8 %)** |
| **Studies with CIRM funding without NIH funding** | | | | |
| 2008 | 10 | 8 (80.0 %) | 7 (70.0 %) | 4 (40.0 %) |
| 2009 | 17 | 12 (70.6 %) | 10 (58.8 %) | 7 (41.2 %) |
| 2010 | 19 | 16 (84.2 %) | 11 (57.9 %) | 5 (26.3 %) |
| 2011 | 29 | 27 (93.1 %) | 22 (75.8 %) | 17 (58.6 %) |
| **Total (2008-2011)** | **75** | **63 (84.0 %)** | **50 (66.7 %)** | **33 (44.0 %)** |
