# Supplementary material for: Power-Laws and the Use of Pluripotent Stem Cell Lines
Source: PLoS One. 2013 Jan 2;8(1):e52068. doi: 10.1371/journal.pone.0052068 (PMC3534668; doi:10.1371/journal.pone.0052068)
Supplement: Table S2 — Use of hESC lines in research papers from Germany. Only cell lines that were used in at least two studies are listed. Numbers in brackets indicate the percentage share of the 56 hESC research papers from Germany published through the end of 2011. Of all the hESC lines that became accessible to German scientists after the amendment of the German Stem Cell Act in 2008, only one (HUES2) was used in more than one published study (note that prior to 2008 use of hESC lines in Germany was restricted in principal to those lines formerly approved by the NIH). In most studies more than one hESC line is used; thus the percentages add up to more than 100. (DOCX) [file pone.0052068.s005.docx]

**Table S2. Use of hESC lines in research papers from Germany.**

Only cell lines that were used in at least two studies are listed. Numbers in brackets indicate the percentage share of the 56 hESC research papers from Germany published through the end of 2011. Of all the hESC lines that became accessible to German scientists after the amendment of the German Stem Cell Act in 2008, only one (HUES2) was used in more than one published study (note that prior to 2008 use of hESC lines in Germany was restricted in principal to those lines formerly approved by the NIH). In most studies more than one hESC line is used; thus the percentages add up to more than 100.

| **Cell Line** | **Numbers of studies involving the hESC line indicated** |
| --- | --- |
| H9 (WiCell) | 36 (64.3 %) |
| H1 (WiCell) | 26 (46.4 %) |
| I3 (Technion) | 6 (10.7 %) |
| HES-3 (ESI, Singapore) | 4 (7.1 %) |
| HES-2 (ESI, Singapore) | 3 (5.4 %) |
| HUES2 (Harvard University) | 2 (3.6 %) |
