# Supplementary material for: Power-Laws and the Use of Pluripotent Stem Cell Lines
Source: PLoS One. 2013 Jan 2;8(1):e52068. doi: 10.1371/journal.pone.0052068 (PMC3534668; doi:10.1371/journal.pone.0052068)
Supplement: Table S3 — Enrichment analysis for formerly NIH approved hESC lines. Statistically significant differences in use of formerly NIH-approved hESC lines by comparison with other hESC lines were identified using hypergeometric enrichment analysis. Significant p-values indicate enrichment of use of the cell lines indicated in the column headers. We observe a highly enriched usage of the formerly NIH approved hESC lines in the US both before and after policy changes in 2009. This pattern is repeated in CIRM funded studies. We note a trend in the CIRM subset towards an increased number of studies using exclusively non eligible lines (i. e. other lines than the formerly approved NIH hESC lines). indicates significant difference; indicates no significant difference. (DOCX) [file pone.0052068.s006.docx]

**Table S3. Enrichment analysis for formerly NIH approved hESC lines.**

Statistically significant differences in use of formerly NIH-approved hESC lines by comparison with other hESC lines were identified using hypergeometric enrichment analysis. Significant *p*-values indicate enrichment of use of the cell lines indicated in the column headers.

We observe a highly enriched usage of the formerly NIH approved hESC lines in the US both before and after policy changes in 2009. This pattern is repeated in CIRM funded studies. We note a trend in the CIRM subset towards an increased number of studies using exclusively non eligible lines (i. e. other lines than the formerly approved NIH hESC lines).

🗹** **indicates significant difference; 🗷** **indicates no significant difference.

| **Subset screened for enrichment and subset compared with** | | **Use of at least one “eligible” hESC line** | **Exclusive use of “eligible” hESC lines** | **Exclusive use of “non-eligible” hESC lines** |
| --- | --- | --- | --- | --- |
| 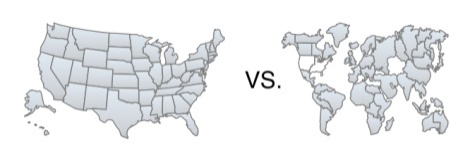 | US vs. rest of the world (all years) | **🗹** p=4.77e-81 | **🗹** p=8.00e-95 | 🗷 |
| 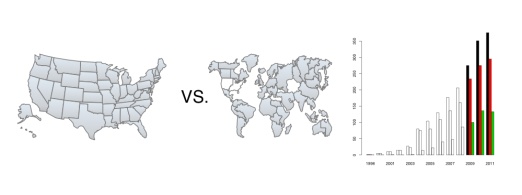 | US vs. rest of the world (since 2009) | **🗹** p=2.55e-54 | **🗹** p=2.26e-60 | 🗷 |
| 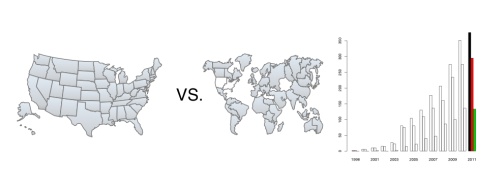 | US vs. rest of the world (2011) | **🗹** p=4.14e-17 | **🗹** p=1.38e-18 | 🗷 |
|  | CIRM vs. other US | **🗷** | **🗷** | 🗷 (p=0.102) |

| **Subset screened for enrichment and subset compared with** | | **Use of at least one “eligible” hESC line** | **Exclusive use of “eligible” hESC lines** | **Exclusive use of “non-eligible” hESC lines** |
| --- | --- | --- | --- | --- |
| 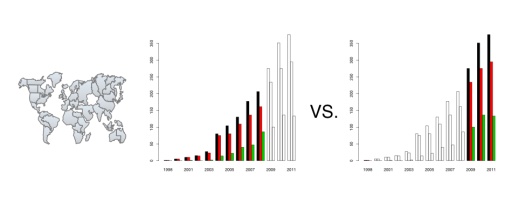 | 2009 and later (global) vs. years before (global) | **🗷** | **🗷** | **🗹** p=0.0025 |
| 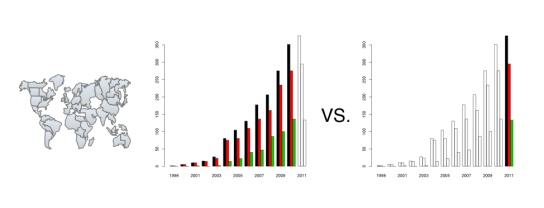 | 2011 (global)  vs. years before (global) | **🗷** | **🗷** | **🗷** (p=0.208) |
| 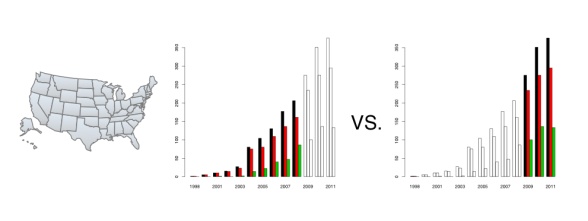 | 2009 and later (US) vs. years before (US) | **🗷** | **🗷** | **🗹** p=0.084 |
| 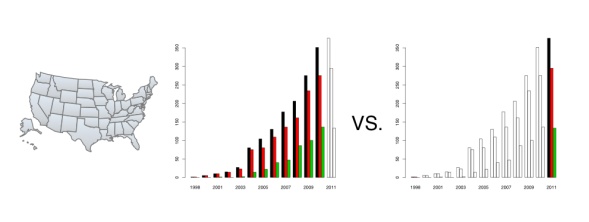 | 2011 (US) vs. years before (US) | **🗷** | **🗷** | **🗹** p=0.072 |
