# Supplementary material for: Power-Laws and the Use of Pluripotent Stem Cell Lines
Source: PLoS One. 2013 Jan 2;8(1):e52068. doi: 10.1371/journal.pone.0052068 (PMC3534668; doi:10.1371/journal.pone.0052068)
Supplement: Table S4 — Characteristics of the seven hESC lines highlighted in Fig. 2C and D . (DOCX) [file pone.0052068.s007.docx]

**Table S4. Characteristics of the seven hESC lines highlighted in Fig. 2C and D.**

| **Cell line** | **Year of first publication of experimental use in a Journal** | **Predicted year of first distribution** | **z-score** | **Innovation/possible explanation** | **Other cell lines from same lab of origin as those highlighted with a z-score > 2** |
| --- | --- | --- | --- | --- | --- |
| BG01 | 2003 | 1998.5 | 2.83 | This cell line was established before August 9th 2001; was eligible for NIH funding; first publication in 2003. | none |
| HUES9 | 2004 | 1999.9 | 3.62 | HUES lines were established to expand the limited set of the eligible NIH lines for researchers with NIH-independent funding; lines were adapted to enzymatic passaging; HUES9 has excellent neural differentiation properties. | HUES7 (2.31), HUES24 (3.14) |
| HSF6 | 2004 | 1999.8 | 3.98 | This cell line was established before August 9th 2001; eligible for NIH funding; first publication in 2003. | none |
| KhES-3 | 2006 | 2001.8 | 5.3 | This cell line was established in Japan and has been preferentially used by Japanese researchers (42 publications total; 39 from Japanese groups, three from UK based groups); adapted to bulk enzymatic passage. KhES3 was first presented in [1]. | KhES-1 (2.27) |
| CA1 | 2007 | 2002.5 | 4.73 | This cell line was established in Canada and has been preferentially used by Canadian researchers (32 publications total; 28 from Canadian groups, two from UK groups, one study each from Israel and Finland); allows for bulk enzymatic passage. The existence of CA1 was publicly announced on 13 June 2005 [2]. | none |
| HS401 | 2008 | 2003.5 | 6.16 | HS401 is a clinical grade hESC line established in 2005 with xeno-free reagents. This cell line has been preferentially used by Scandinavian groups (20 publications total; 7 from groups based in Finland, 6 from Swedish groups, three from Swiss groups, one study each from Austria, Czechoslovakia, Spain and UK based groups). The existence of HS401 was publicly announced in 2006 [3]. | HS293 (4.93), HS346 (2.21), HS360 (2.21) |
| WIBR3 | 2010 | 2005.7 | 7.53 | WIBR3 is a female hESC line established under 5% oxygen conditions, possibly representing a more naïve pluripotent state that potentially can be monitored through its X-inactivation state. | none |

**Supplementary References**

1. Nakatsuji N (2005) Establishment and manipulation of monkey and human embryonic stem cell lines for biomedical research. Ernst Schering Res Found Workshop: 15-26.

2. Wong J (2005) Canadian Human Embryonic Stem Cell Lines Developed. The Bulletin, University of Toronto 58: 6.

3. Hovatta O (2006) Derivation of human embryonic stem cell lines, towards clinical quality. Reprod Fertil Dev 18: 823-828.
